# Supplementary material for: Splice-Junction-Based Mapping of Alternative Isoforms in the Human Proteome
Source: Cell Rep. Author manuscript; Available in PMC 2020 Jan 15. (PMC6961840; doi:10.1016/j.celrep.2019.11.026)
Supplement: 2 [file NIHMS1546469-supplement-2.zip › DF1/PXD009737/TestisTrypsin/TestisTrypsin_18_PDZD2_IGRPTVLLGSSAVIR.pdf]

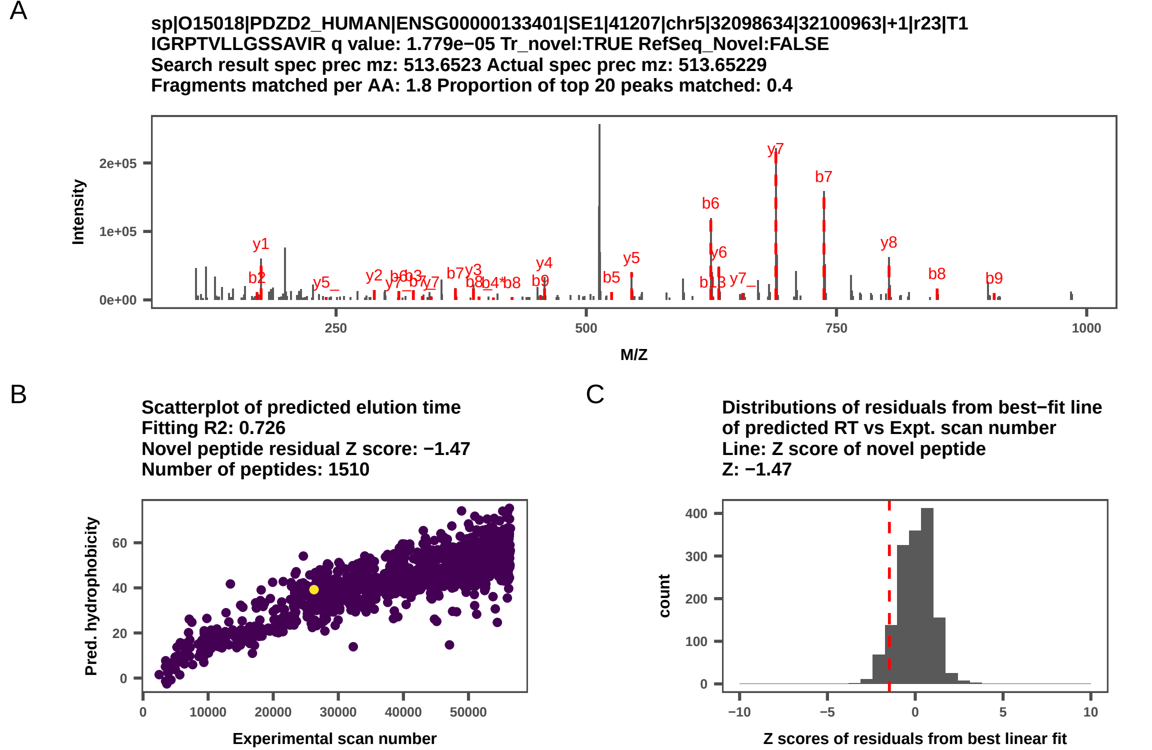
A
sp|O15018|PDZD2_HUMAN|ENSG00000133401|SE1|41207|chr5|32098634|32100963|+1|r23|T1
IGRPTVLLGSSAVIR q value: 1.779e−05 Tr_novel:TRUE RefSeq_Novel:FALSE
Search result spec prec mz: 513.6523 Actual spec prec mz: 513.65229
Fragments matched per AA: 1.8 Proportion of top 20 peaks matched: 0.4
Intensity
M/Z
B
Scatterplot of predicted elution time
Fitting R2: 0.726
Novel peptide residual Z score: −1.47
Number of peptides: 1510
Pred. hydrophobicity
Experimental scan number
C
Distributions of residuals from best−fit line
of predicted RT vs Expt. scan number
Line: Z score of novel peptide
Z: −1.47
count
Z scores of residuals from best linear fit
